# Analysis of long non-coding RNA expression profiles in high-glucose treated vascular endothelial cells

**DOI:** 10.1186/s12902-020-00593-6

**Published:** 2020-07-20

**Authors:** Erqin Xu, Xiaolei Hu, Xiaoli Li, Guoxi Jin, Langen Zhuang, Qiong Wang, Xiaoyan Pei

**Affiliations:** 1 Room of Physical Diagnostics, Clinical College of Medicine, Bengbu Medical College, Bengbu, Anhui 233030 P.R. China; 2 Department of Endocrinology, First Affiliated Hospital of Bengbu Medical College, 287 Changhuai Road, Longzihu Zone, Bengbu, Anhui Province 233004 People’s Republic of China

**Keywords:** lncRNA, Diabetes mellitus, HUVECs, Expression profiling, Hyperglycemia, RNA-seq

## Abstract

**Background:**

Diabetes mellitus is often associated with microvascular and macrovascular lesions, and hyperglycemia-induced vascular endothelial cell damage is a key factor.

**Methods:**

We investigated long non-coding RNAs (lncRNAs) and mRNAs that are affected by hyperglycemia-induced damage using human umbilical vein endothelial cells (HUVECs) as a model. HUVECs were cultured under high (25 mmol/L) or normal (5 mmol/L) glucose conditions for 6 d, and then lncRNAs and protein-coding transcripts were profiled by RNA-seq.

**Result:**

Among 40,379 lncRNAs screened, 214 were upregulated (log2 [fold-change] > 1, FDR < 0.05) and 197 were downregulated (log2 [fold-change] < − 1, FDR < 0.05) in response to high-glucose. Furthermore, among 28,431 protein-coding genes screened, 778 were upregulated and 998 were downregulated. A total of 945 lncRNA/mRNA pairs were identified, including 126 differentially expressed lncRNAs predicted to target 201 mRNAs, among which 26 were cis-regulatory interactions. The corresponding lncRNA-mRNA network was composed of 354 lncRNA nodes, 1167 mRNA nodes and 9735 edges. Dozens of lncRNAs with high degree may play important roles in high-glucose-induced HUVEC damage, including ENST00000600527, NONHSAT037576.2, NONHSAT135706.2, ENST00000602127, NONHSAT200243.1, NONHSAT217282.1, NONHSAT176260.1, NONHSAT199075.1, NONHSAT067063.2, NONHSAT058417.2.

**Conclusion:**

These observations may provide novel insights into the regulatory molecules and pathways of hyperglycemia-related endothelial dysfunction in diabetes-associated vascular disease.

## Background

Diabetes-related microvascular and macrovascular complications are directly correlated with the severity and duration of hyperglycemia [1, 2]. Stable and healthy endothelial cells are the basis of normal blood vessels, whereas dysfunction of endothelial cells is a risk indicator of diabetic angiopathy [3, 4]. Protein kinase C, hexosamine pathways, polyol pathways, and advanced glycation end-products are believed to be responsible for dysfunction associated with endothelial cell diabetes mellitus. These factors inhibit the production of nitric oxide by promoting the bioavailability of reactive oxygen species, thus altering the structure and physiology of endothelial cells [4–8]. Hyperglycemia is the main contributor of endothelial dysfunction [9]. Advanced glycation end products of hyperglycemia compromise the bioavailability of nitric oxide, which essentially drives endothelial dysfunction [10]. The effect of advanced glycation end products on monocytes, macrophages and vascular smooth muscle cells enhances the inflammatory response and oxidative stress in the endothelial system [9, 10]. Thus, unlike other cells or tissues, the vascular endothelium is extremely sensitive to blood glucose and is the direct target of hyperglycemia injury [11].

Long non-coding RNAs (lncRNAs) are non-coding RNA molecules that contain over 200 nucleotides. LncRNAs play important regulatory roles in various diseases, such as cancers [12–15], atherosclerosis [15], neurodegeneration [11], autoimmune disorders [16–20], and chronic vascular disease [21]. Data from both in vitro hyperglycemia induction and in vivo diabetes mellitus experiments have shown that lncRNA MALAT1 is highly expressed, and that inhibition of its expression can effectively improve endothelial cell inflammation and diabetic retinopathy [22, 23]. Furthermore, high glucose (HG) treatment of vascular endothelial cells is accompanied by increased expression of lncRNA MIAT, and interference with MIAT can promote the proliferation, migration and abnormal angiogenesis of vascular endothelial cells induced by HG [24]. In human endothelial cells, the knockdown of lncRNA AGAP2-AS1 inhibits cell proliferation, tubule formation and acetylated LDL uptake [25]. Additionally, HG or oxidative stress inhibits the expression of lncRNA MEG3 in endothelial cells and diabetic mice. Knockout of lncRNA MEG3 can aggravate retinal vascular dysfunction and increase microvascular leakage and inflammation [26]. These results suggest that lncRNAs may comprise novel therapeutic targets in hyperglycemia-related endothelial dysfunction or diabetes-induced vascular disease. In order to further understand the role of lncRNA in endothelial cell injury in diabetes mellitus, we carried out high throughput sequencing using human umbilical vein endothelial cells (HUVECs) cultured under normal (CN) or HG conditions.

## Methods

### Cell culture

HUVECs were purchased from the China Center for Type Culture Collection (CCTCC, Wuhan, China), and were maintained in Modified Eagle’s Medium (MEM; Hyclone/Thermo Fisher Scientific, Waltham, MA, USA) supplemented with 10% fetal bovine serum (FBS; Gibco, Gaithersburg, MD USA), penicillin (100 U/mL; Gibco), and streptomycin (100 μg/mL; Gibco) at 37 °C in a humidified 5% CO_2_ chamber. Confluent HUVECs were starved overnight before exposure to media with normal or HG. HUVECs assigned to the Control (CN) group were maintained for 6 d in 5 mmol/L glucose and 20 mmol/L mannitol to account for changes that may be triggered by osmolarity differences. The remaining HUVECs of the HG group were maintained for the same duration in glucose-enriched (final glucose concentration 25 mmol/L) media. There were three biological duplicates in each group of HUVECs.

### RNA preparation and RNA-seq

TRIzol reagent (Invitrogen, Carlsbad, CA, USA) was used to isolate and purify total RNA according to the manufacturer’s instructions. The quantity and quality of the RNA were determined using a NanoDrop 2000 instrument (Thermo, Fisher Scientific). The RNA integrity was assessed by electrophoresis with denaturing agarose gels. Libraries were constructed according to the standard TruSeq protocol. Sequencing was performed on the Illumina HiSeq 2500 according to the manufacturer’s protocol at Ao-Ji Biotech (Shanghai, China).

### Identification of lncRNAs by using a computational approach

Quality control of the RNA-Seq reads was conducted using FastQC (v0.11.3). Reads were trimmed using the software SEQTK for known Illumina TruSeq adapter sequences, poor reads and ribosome RNA reads. Trimmed reads were aligned to homo sapiens genome (hg38) using Hisat2 (version: 2.0.4) [27]. Transcripts were assembled using Stringtie (v1.3.0) and then were compiled together by gffcompare (v0.9.8) [28, 29], Transcripts with class codes “i,” “u,” and “x,” were considered to be potential novel long transcripts. Pfam [30], CPC [31] and CNCI [32] were used to compute the coding potential of each novel transcript. Transcripts with a Pfam score < 0, CNCI < 0 and CPC non-significant were considered to lack coding potential. Transcripts were matched with annotation databases, including NONCODE (v5) (http://www.noncode.org) [33] and Ensembl [34] The matched transcripts were considered to be known lncRNAs, and others were considered to be novel lncRNAs. All lncRNAs were quantified using Stringtie. According to the positional association between lncRNA and mRNA in the genome, lncRNA was classified into six types: Bidirectional, exonic_antisense, exonic_sense, intergenic, intronic_antisense and intronic_sense [35].

### Prediction and functional analysis of target genes of differentially expressed lncRNAs

Chromosome localizations, sequence complementarity and correlation coefficients between lncRNA and mRNA pairs were analyzed in order to identify the lncRNAs’ cis- and trans-target genes. In brief, a distinction was made according to chromosome location: if lncRNAs were located in the range of 10 KB upstream and 20 KB downstream of the coding gene, they were considered likely to be cis-regulatory [26, 27]. The trans-regulatory interaction potential between lncRNAs and mRNAs was analyzed by RNAplex software [28], with binding energy of <− 30 as the threshold. Furthermore, the Pearson correlation coefficients (PCCs) between lncRNAs and mRNAs were calculated, with a cutoff of PCC ≥ 0.6. The functions of these candidate coding genes were assessed using gene ontology (GO) function and pathway analysis using ClusterProfiler.

### The lncRNA-mRNA-coexpression network

The lncRNA and mRNA co-expression network was constructed according to the normalized fragments per kilobase of transcript per million mapped reads of the unit genes. We calculated the PCC between differentially expressed lncRNAs and differentially expressed mRNAs. LncRNA-mRNA pairs with significant correlations (PCC > 0.99 and *p* < 0.01) were chosen to build the co-expression network and visualized by Cytoscape. The number of directly linked neighbors for each node was calculated and was defined as the nodes degree.

### RT-PCR validation

Seven lncRNAs were randomly selected for verification of the RNA-Seq results by quantitative real-time PCR (qRT-PCR), which was performed on a Roche LightCycler 480 machine (Roche Applied Science, Germany) with the SYBR green assay (TaKaRa, Japan). The qRT-PCR amplification reactions were carried out via the following program: 95 °C for 10 min, 40 cycles with 95 °C for 15 s and 60 °C for 20 s. The primer sequences for qRT-PCR are provided in Table 1. With GAPDH as an internal control, the relative expression was computed according to the 2^−ΔΔCt^ method.

**Table 1:** Sequences of primers used in this study

Gene	Primers sequences	PCR product length (bp)
GAPDH	CCTGGTATGACAACGAATTTG	131
	CAGTGAGGGTCTCTCTCTTCC	
ENST00000444438	AGGTGTGTGTCAATCCCAACT	146
	ACTTGCTGCTCGTCCTTTCT	
ENST00000623851	CCTCCACCCACAGACATCTT	111
	TCCACTCCTCTGGTGTCCTG	
NONHSAT108582.2	CTGGGGCCTTTTCACTCCTT	99
	GTTTTTCCCTGTCCCGGCTA	
NONHSAT141593.2	AAACAAGCTGCCTCCAACCT	115
	GGAGCAAAAAGCTGCTCTCG	
ENST00000601562	GCTTCCGTTCGCTTGACTG	85
	ACAACCGATTTTGCTCTGCG	
ENST00000609170	ATGGATGCCTTGGGGACTCT	96
	GCACTACCGGTGGGATTTCA	

## Results

### LncRNA-sequencing data analysis

**Fig. 1:**
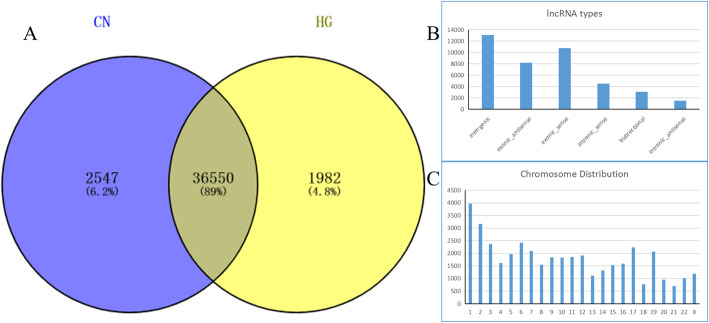
The landscape of lncRNAs identified in the Control Normal (CN) and High Glucose (HG) groups. **a** Venn diagram of lncRNAs in the CN and HG groups. **b** Number of lncRNAs classified into each of six types. **c** Number of lncRNAs on each chromosome

We characterized the lncRNA landscape of expression by performing deep RNA-seq experiments on three CN and three HG-induced HUVECs samples. After SEQTK quality assessment, more than 33 million total original reads for each sample were obtained, and the proportion of bases with quality values greater than 20 (Q20) was > 94%. These results indicate that the quality of the sequencing results was acceptable (Table 2). After filtering out the adaptor sequence and low quality reads, the percentage of clean reads within the raw reads accounted for 94% of the total sequences in the two groups. Hisat2 software was used to map the clean reads to * homo sapiens* reference genome. As shown in Table 2, approximately 97% of the trimmed reads were mapped onto the reference genome. In total, we screened 40,380 lncRNAs from the six samples, including 387 novel and 39,993 known lncRNAs, of which 36,550 were shared lncRNAs detected in both the HG and CN HUVEC groups (Fig. 1a, Supplemental Table S1). Most of the identified lncRNAs were transcribed from protein-coding exons (sense and antisense); others were from introns and intergenic regions (Fig. 1b). Furthermore, 24,304 lncRNA transcripts could be found in all chromosomes, with the majority located on chromosome 1 (Fig. 1c).

**Table 2:** Quality control results for Control (CN) and High Glucose (HG) samples

Sample ID	Raw reads	Clean reads	Clean ratio	rRNA trimmed	rRNA ratio	No rRNA pair
CN1	117,704,886	113,050,654	96.05%	112,662,073	0.34%	110,698,298
CN2	122,274,264	117,706,060	96.26%	116,809,617	0.76%	114,445,122
CN3	133,367,858	128,173,211	96.11%	127,454,102	0.56%	125,334,594
HG1	131,986,802	127,345,509	96.48%	126,055,444	1.01%	123,898,326
HG2	125,081,888	120,021,621	95.95%	118,751,346	1.06%	116,639,846
HG3	119,510,382	114,549,751	95.85%	113,422,883	0.98%	111,534,600

### Identification of differentially expressed lncRNAs

**Fig. 2:**
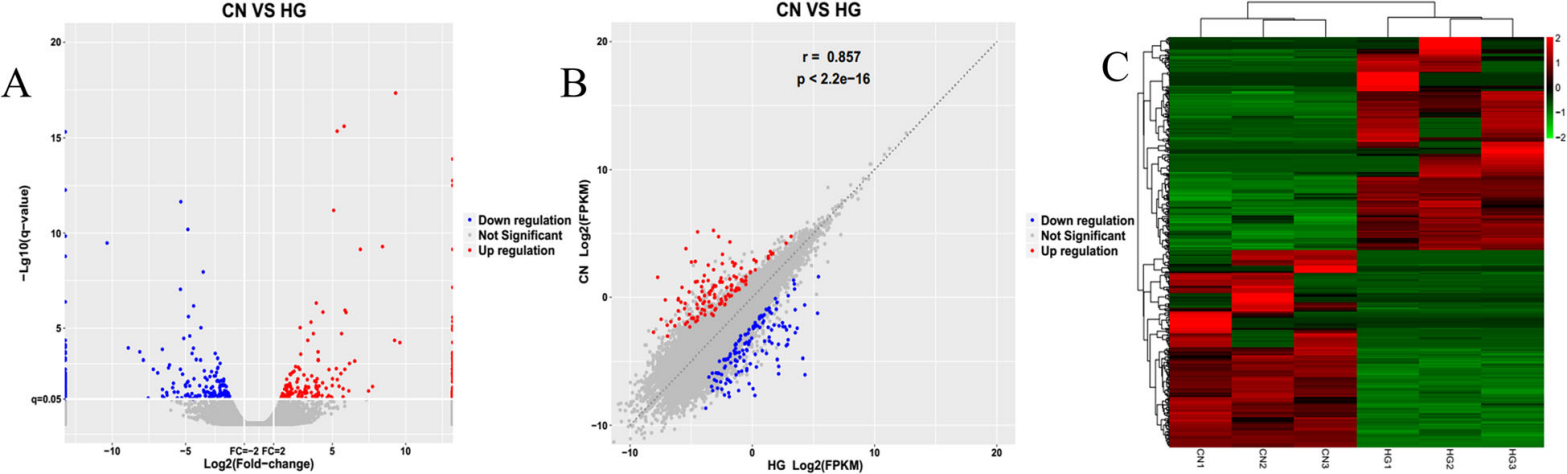
Characterization of differentially expressed lncRNAs in High Glucose (HG) versus Control Normal (CN) cells. **a** Correlation plots, **b** volcano plots, and **c** heatmap of differentially expressed lncRNAs. For panels **a** and **b**, blue indicates > 2 fold decreased expression and red indicates > 2 fold increased expression of the dysregulated lncRNAs in HG-induced human umbilical vein endothelial cells (HUVECs) (*p* < 0.05). The gray indicates no significant change. For panel C, red indicates high expression and green indicates low expression of genes with > 2 fold dysregulation for each of the 6 samples

EdgeR was used to filter differentially expressed lncRNAs (DELs) between the HG-induced and CN HUVEC groups. Among the lncRNAs, 214 were significantly upregulated (log2 (fold-change) > 1, FDR < 0.05) and 197 were significantly downregulated (log2 (fold-change) < − 1, FDR < 0.05) in response to HG exposure (Fig. 2). Additionally, several of the DELs had a fold change value equal to positive infinity and negative infinity, meaning that these lncRNAs were completely switched-on or off with HG induction. The top five upregulated DELs were NONHSAT180405.1, MSTRG.31780.5, NONHSAT086922.2, NONHSAT022138.2, NONHSAT094345.2 and the top five downregulated DELs were NONHSAT056661.2, NONHSAT204850.1, NONHSAT217441.1, MSTRG.9798.2, NONHSAT152502.1 (Supplemental Table S2).

### qRT-PCR verification of DELs

**Fig. 3:**
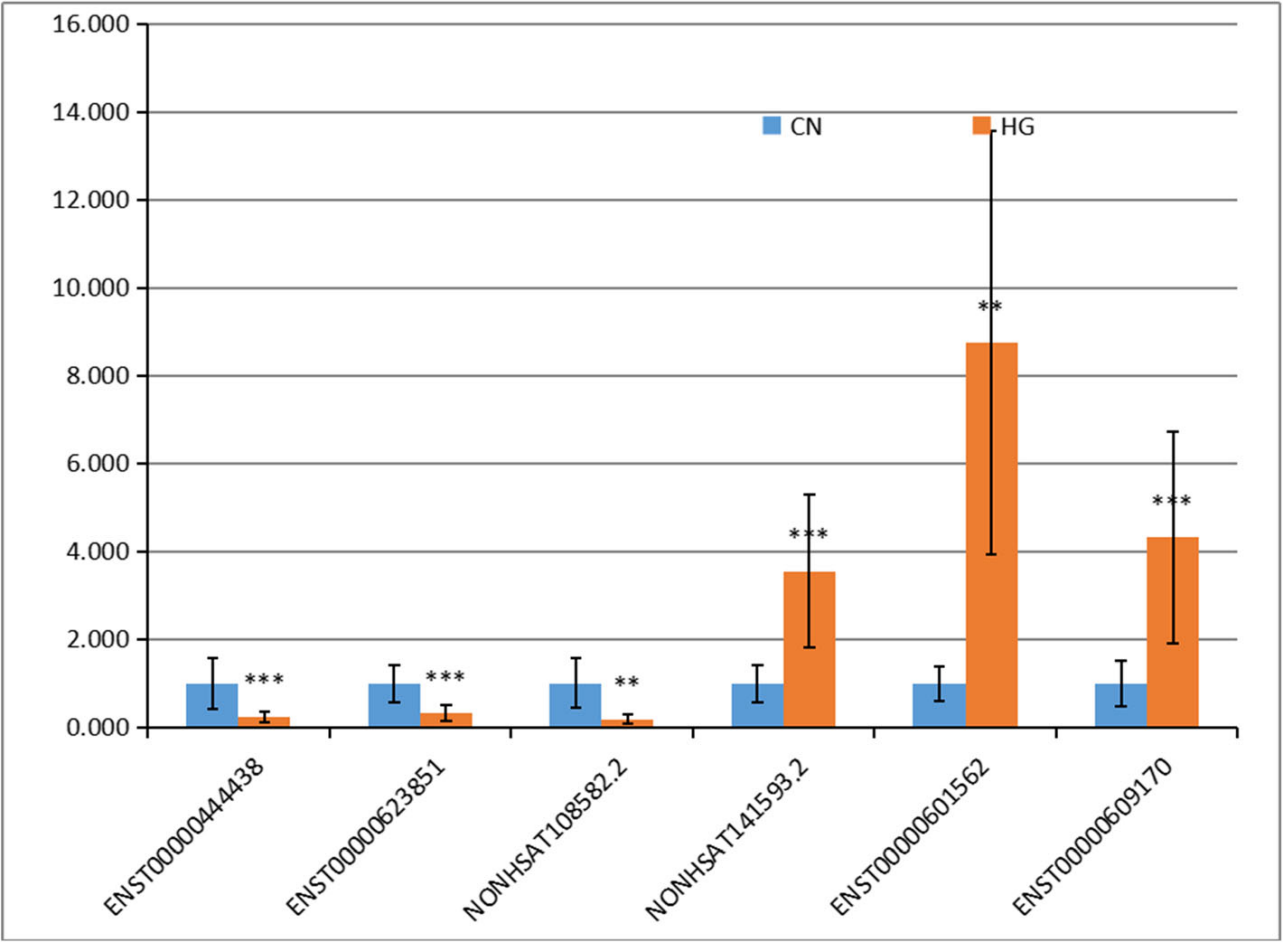
Verification of differentially expressed lncRNAs (DELs) by qRT-PCR. The expression of six lncRNAs in human umbilical vein endothelial cells (HUVECs) was detected by qRT-PCR, Results indicate the expression fold changes relative to that of the Control Normal (CN) sample (1.0)

To verify our findings, the expression profiles of six differentially expressed lncRNAs were randomly selected for qRT-PCR analysis. There were three repeats per group and five repeats per sample in the qPCR. The results show that the expression of the lncRNAs had similar trends as with the sequencing results, indicating that our sequencing results were reliable (Fig. 3).

### Regulatory analysis of DELs and DEGs

lncRNAs act via cis- and trans-regulation of target genes for biological function. To evaluate the regulatory pathways associated with the lncRNAs, we assessed the differentially expressed genes (DEGs) in the same HUVEC samples. Of 28,431 protein-coding genes that were screened, 778 were upregulated and 998 downregulated by HG treatment. By comparing the DELs and the DEGs, a total of 945 matched lncRNA-mRNAs pairs for 126 DELs and 201 DEGs were predicted, of which 26 lncRNA/mRNA interactions were cis-regulatory, with either positive or negative correlations of the lncRNAs with their predicted target genes. An additional 715 interactions were trans-regulatory, including 2 that were both cis- and trans-regulatory (Supplemental Table S3).

**Fig. 4:**
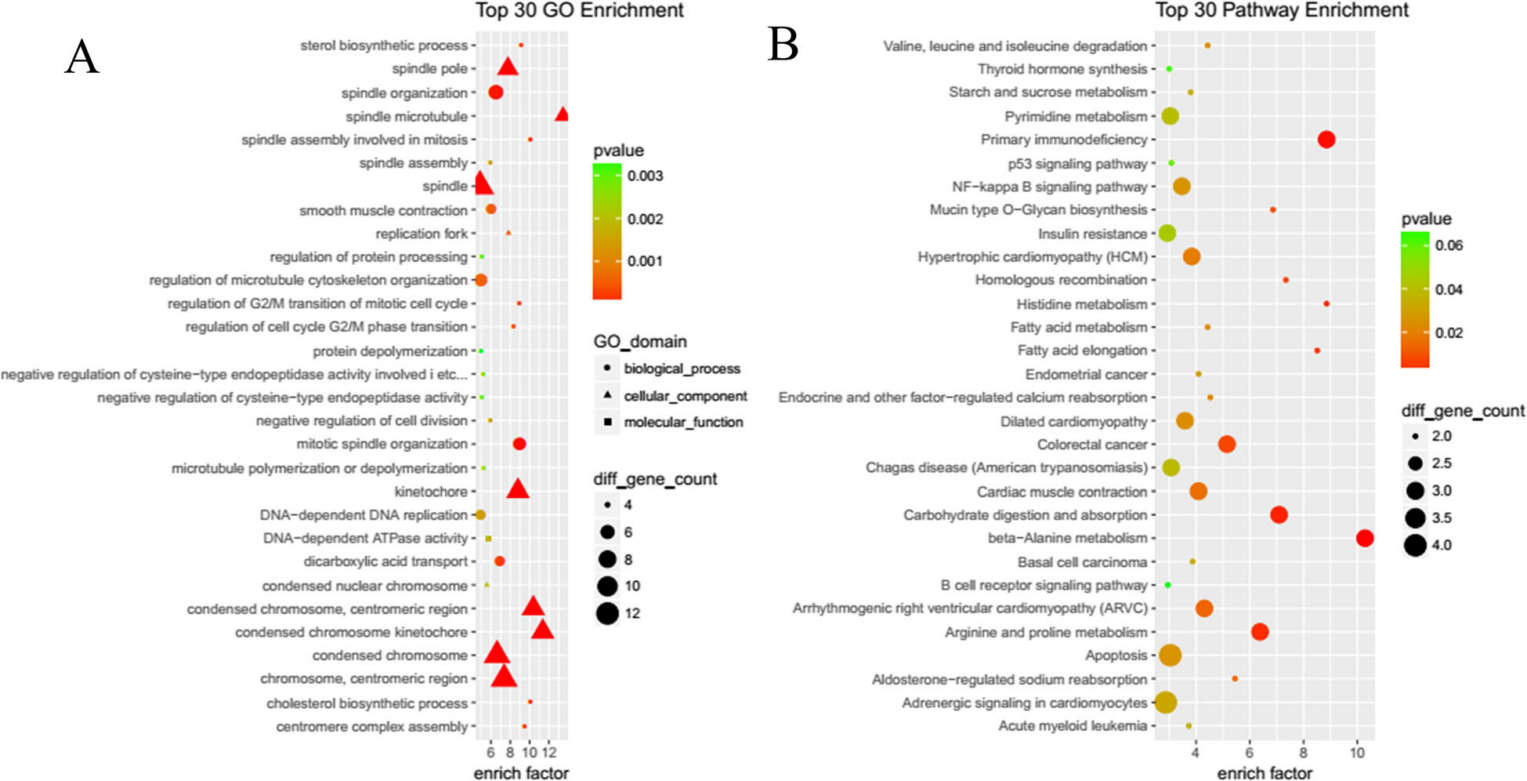
Gene Ontology (GO) and KEGG pathway enrichment analysis for cis- and trans-target genes of differentially expressed lncRNAs (DELs). **a** Top30 GO enrichment terms. **b** Top30 KEGG pathway enrichment terms

To further understand the regulatory functions of the differentially expressed lncRNAs, all predicted target genes were annotated according to GO and pathway function entries using ClusterProfiler. Among the GO Enrichment terms (Fig. 4a) the most abundant in the biological process categories were Mitotic cell cycle, Cell cycle, Cell division, Microtubule cytoskeleton organization, DNA replication, Chromosome segregation, Spindle organization, Cytoskeleton organization, Cholesterol biosynthetic process, and Centromere complex assembly. The most abundant GO terms in the cellular component categories were, Molecular function Centromeric region, Chromosome, spindle, Chromosome, Replication fork, Nuclear chromosome, Condensed nuclear chromosome, Microtubule, Microtubule cytoskeleton, Cytoskeleton, and Nucleoplasm. Among the Pathway Enrichment terms (Fig. 4b), the most abundant were beta-Alanine metabolism, Primary immunodeficiency, Carbohydrate digestion and absorption, Arginine and proline metabolism, Histidine metabolism, Fatty acid elongation, Homologous recombination, Colorectal cancer, Mucin type O-Glycan biosynthesis, Arrhythmogenic right ventricular cardiomyopathy (ARVC), Aldosterone-regulated sodium reabsorption, Cardiac muscle contraction, Hypertrophic cardiomyopathy (HCM), Endocrine and other factor-regulated calcium reabsorption, Dilated cardiomyopathy, Valine, leucine and isoleucine degradation, Fatty acid metabolism, Apoptosis, NF-kappa B signaling pathway, Endometrial cancer, Adrenergic signaling in cardiomyocytes, and Hippo signaling pathway.

### lncRNA-mRNA co-expression network

**Fig. 5:**
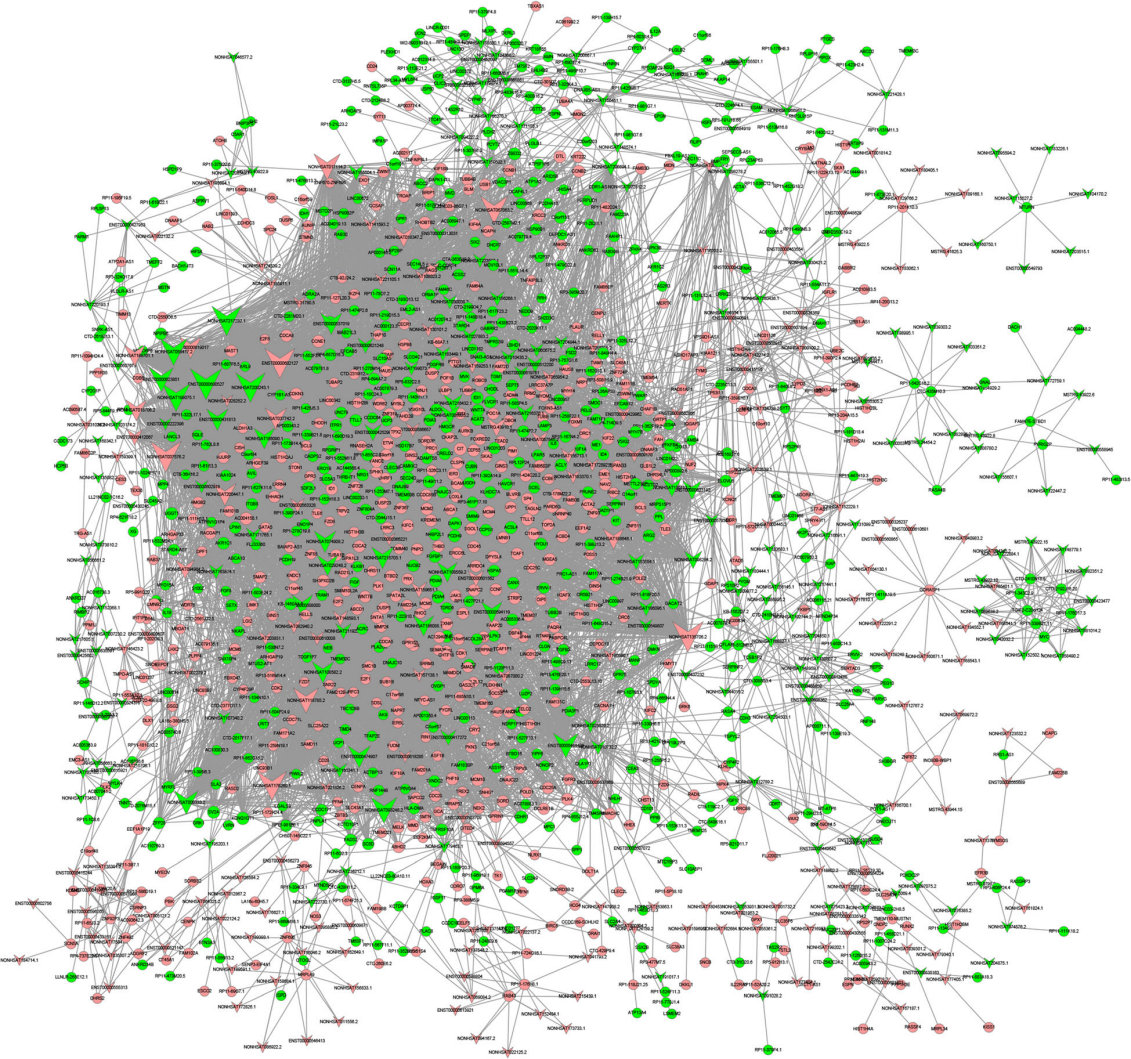
A lncRNA-gene-network based on Pearson’s correlation coefficient analysis of differentially expressed lncRNAs (DELs) from High Glucose (HG) versus Control Normal (CN) human umbilical vein endothelial cells (HUVECs). Pink nodes indicate upregulated mRNAs or lncRNAs, and green nodes indicate downregulated mRNAs or lncRNAs

**Fig. 6:**
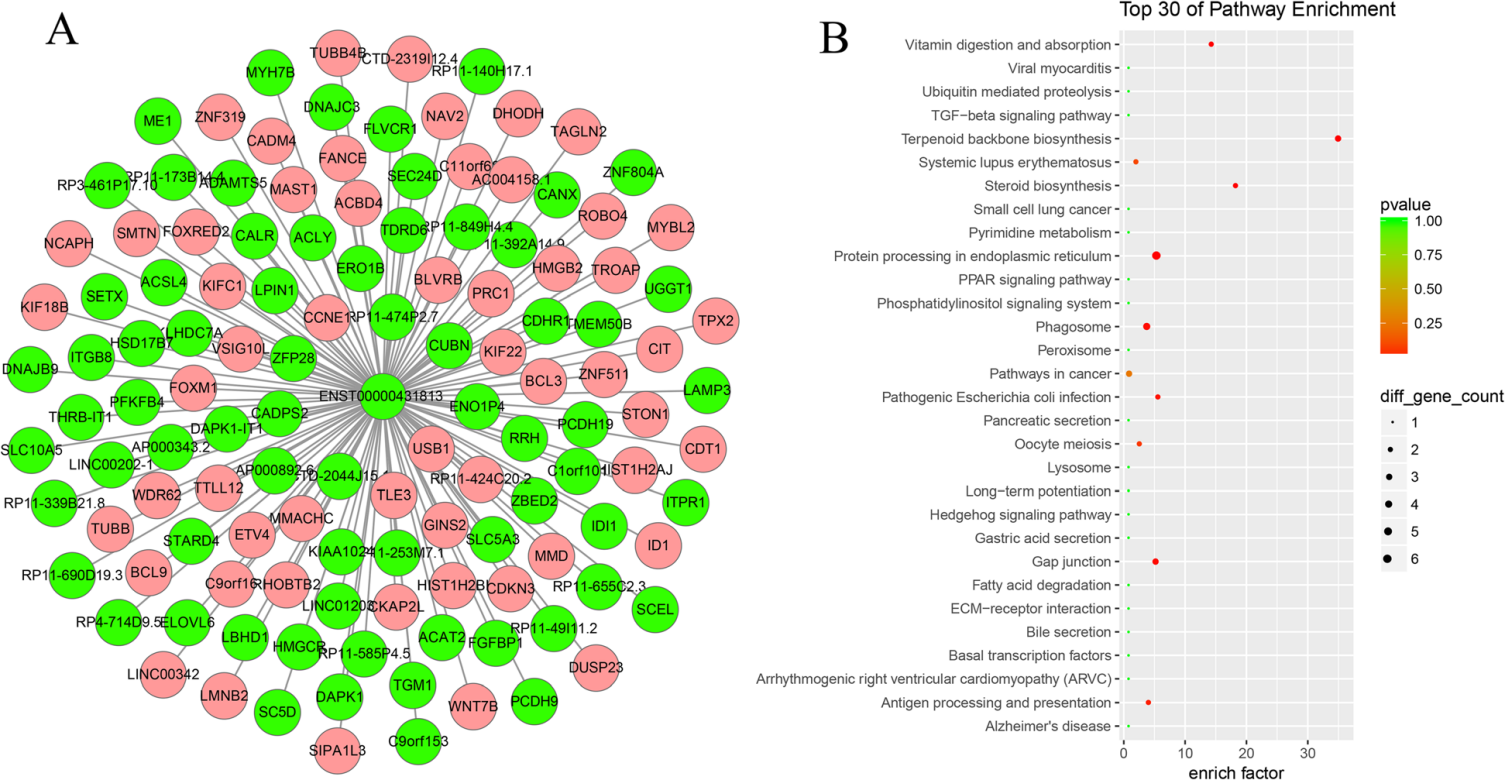
Interaction between LncRNA DAPK1-IT1 and differentially expressed mRNAs. **a** The network of ENST00000431813 (DAPK1-IT1) and mRNAs. **b** The enrichment of KEGG pathway of ENST00000431813. Pink nodes indicate upregulated mRNAs, and green nodes indicate downregulated mRNAs or lncRNAs

To visualize the co-expression network, pairs of lncRNAs and mRNAs that had PCC > 0.99 and *p* < 0.01 were assessed using Cytoscape software. As shown in Fig. 5, the lncRNA–mRNA network was composed of 354 lncRNA nodes, 1167 mRNA nodes and 9735 edges. According to the nodes and connections, the top 10 LncRNAs that could connect with highest protein coding genes were ENST00000600527 (degree = 241), NONHSAT037576.2 (degree = 234), NONHSAT135706.2 (degree = 233), ENST00000602127 (degree = 226), NONHSAT200243.1 (degree = 221), NONHSAT217282.1 (degree = 219), NONHSAT176260.1 (degree = 216), NONHSAT199075.1 (degree = 204), NONHSAT067063.2 (degree = 197), and NONHSAT058417.2 (degree = 192) (Fig. 5). In our analysis, we found some famous lncRNAs, which could connect with more than 100 protein coding genes, such as: MUC20-OT1 (ENST00000600527 and ENST00000602127), TIMM23B-AGAP6 (ENST00000444438), DAPK1-IT1 (ENST00000431813), and ZNF528-AS1 (ENST00000594119). Taking ENST00000431813 (DAPK1-IT1) as an example, we constructed a network diagram of its interaction with mRNA (Fig. 6).

## Discussion

Using microarray analysis, a variety of lncRNAs have been determined to be dysregulated in HG-induced HUVECs [36]. In addition, mechanistic studies indicate a role for specific lncRNAs in endothelial cell dysfunction induced by diabetes or HG [22–26]. Both clinical and experimental studies indicate that impairment of vascular smooth muscle cells by diabetes and HG contribute to the increased incidence of diabetic cardiomyopathy [37]. Furthermore, a large number of studies have shown that lncRNAs are involved in the injury of vascular smooth muscle induced by diabetes mellitus. For example, lncRNA-ES3 can regulate calcification/senescence of vascular smooth muscle cells through an miR-34c-5p/BMF axis that is activated upon HG induction [38]. The lncRNA SENCR shows high abundance of expression in vascular cells [37, 39, 40], and can regulate fork-head box protein O1 and transient receptor potential cation channel 6, thereby promoting the proliferation and migration of smooth muscle cells. HG exposure of T2DM db/db mice can inhibit the expression and function in smooth muscle cells, and overexpression of SENCR reverses the inhibitory effect of HG on vascular smooth muscle cells [40]. Given the important role of these lncRNAs, we rationalized that it is likely that other uncharacterized lncRNAs participate in the processes of endothelial cell pathogenesis caused by diabetes. Further, RNA-seq—as a highly sensitive approach towards identifying differentially expressed RNAs—could be used for the purpose of identifying the roles of these yet uncharacterized lncRNAs.

In the present study, a total of 214 upregulated and 197 downregulated lncRNAs (|FC| > 2, FDR < 0.05) were identified through RNA-seq using an HG-induced HUVEC model. Quality control assays were performed to ensure the reliability of the experiments, and several lncRNAs were validated using RT-qPCR. Furthermore, DEGs in the same cells were identified, and the lncRNA and mRNA expression profiles were compared. Using a variety of bioinformatics approaches, we revealed pathways and processes associated with dysregulation of lncRNA expression. It is well known that the AGE-RAGE signaling pathway in diabetic complications plays an important role in endothelial cell injury induced by diabetes mellitus. In Enrichment Analysis based on lncRNA-mRNA co-expression networks, up to 45 lncRNAs were found to be associated with this pathway, especially ENST00000600527, NONHSAT217282.1, NONHSAT067063.2, NONHSAT093248.2, NONHSAT118785.2, ENST00000444438, NONHSAT200447.1, NONHSAT108582.2, ENST00000508000, ENST00000594119, NONHSAT186088.1. Therefore, our findings are consistent with current understanding of processes by which diabetes promotes endothelial cell injury. On the basis of our findings, the information obtained in this study should be useful for identifying additional pathways and processes that contribute to the pathogenesis of diabetes.

Moreover, we identified some known lncRNAs may participate in high-glucose treated HUVEC. DAPK1-IT1, DAPK1 intronic transcript 1, has proved involved in cancer progression [41, 42], chemotherapy insensitivity [43], Atherogenesis [44, 45], etc. In our study, we constructed the coexpression subnet of DAPK1-IT1 and function was analyzed with KEGG pathway (Fig. 6), found may function with Terpenoid backbone biosynthesis, Steroid biosynthesis, Vitamin digestion and absorption, Protein processing in endoplasmic reticulum, Phagosome, and some of these pathways have been proved to be involved in the occurrence and development of diabetes. LINC00969 was identified as a competitive endogenous RNA (CERNA) of miR-335-3p in vitro, which positively regulates the expression of Thioredoxin interacting protein (TXNIP) [46]. We will conduct further clinical studies on other lincRNAs in the future.

## Conclusion

The potential roles of lncRNAs in diabetic complications were investigated by bioinformatics analysis. These findings may help us to understand the possible molecular mechanism of HG-induced HUVECs and may provide a more comprehensive understanding of the lncRNA expression profile that is dysregulated during diabetes.

## Supplementary information

**Additional file 1: Table S1**. List of 40,380 lncRNAs screened from HG and CN HUVEC groups.

**Additional file 2: Table S2**. The top upregulated and downregulated DELs.

**Additional file 3: Table S3**. Expression correlations of 715 lncRNA-mRNAs trans-regulatory pairs.

## Data Availability

The sequences can be accessed after (1 May 2021) at (https://dataview.ncbi.nlm.nih.gov/object/PRJNA534362?reviewer=kbilbcamsojq6imld1g394slf2) with (PRJNA534362). Until then, the sequences are available from the corresponding author upon reasonable request.

## Abbreviations

lncRNAs: Long non-coding RNAs
HUVECs: Human umbilical vein endothelial cells
HG: High glucose
GO: Gene ontology
ARVC: Arrhythmogenic right ventricular cardiomyopathy
